# Evidence for Plastic Processes in Migraine with Aura: A Diffusion Weighted MRI Study

**DOI:** 10.3389/fnana.2017.00138

**Published:** 2018-01-17

**Authors:** Nikoletta Szabó, Péter Faragó, András Király, Dániel Veréb, Gergő Csete, Eszter Tóth, Krisztián Kocsis, Bálint Kincses, Bernadett Tuka, Árpád Párdutz, Délia Szok, János Tajti, László Vécsei, Zsigmond T. Kincses

**Affiliations:** ^1^Neuroimaging Research Group, Department of Neurology, Albert Szent-Györgyi Clinical Center, University of Szeged, Szeged, Hungary; ^2^Central European Institute of Technology, Brno, Czechia; ^3^MTA-SZTE Neuroscience Research Group, Szeged, Hungary

**Keywords:** DTI, microstructure, neurodegeneration, plasticity, TBSS, white matter

## Abstract

**Background:** Formerly white matter abnormalities in a mixed group of migraine patients with and without aura were shown. Here, we aimed to explore white matter alterations in a homogeneous group of migraineurs with aura and to delineate possible relationships between white matter changes and clinical variables.

**Methods:** Eighteen patients with aura, 25 migraine patients without aura and 28 controls were scanned on a 1.5T MRI scanner. Diffusivity parameters of the white matter were estimated and compared between patients’ groups and controls using whole-brain tract-based spatial statistics.

**Results:** Decreased radial diffusivity (*p* < 0.036) was found bilaterally in the parieto-occipital white matter, the corpus callosum, and the cingular white matter of migraine with aura (MwA) patients compared to controls. Migraine without aura (MwoA) patients showed no alteration compared to controls. MwA compared to MwoA showed increased fractional anisotropy (*p* < 0.048) in the left parieto-occipital white matter. In MwA a negative correlation was found between axial diffusivity and disease duration in the left superior longitudinal fascicle (left parieto-occipital region) and in the left corticospinal tract (*p* < 0.036) and with the number of the attacks in the right superior longitudinal fascicle (*p* < 0.048).

**Conclusion:** We showed for the first time that there are white matter microstructural differences between these two subgroups of migraine and hence it is important to handle the two groups separately in further researches. We propose that degenerative and maladaptive plastic changes coexist in the disease and the diffusion profile is a result of these processes.

## Introduction

In 20% of cases, migraine is preceded by focal neurological symptoms, such as visual, sensory, motor or verbal disturbances, called aura. This special form of migraine [MwA (Lauritzen, 1994)] starts with a different trigger than MwoA. CSD, the probable electrophysiological correlate for the aura phenomenon, is thought to occur exclusively in MwA (Lauritzen, 1994; Hadjikhani et al., 2001). Debate continues whether MwA and MwoA are separate entities; some studies question the viability of a distinction between them (Ranson et al., 1991; Manzoni and Torelli, 2008).

Imaging studies revealed that migraine is associated with structural changes affecting gray (Kim et al., 2008; Schmidt-Wilcke et al., 2008; Schmitz et al., 2008; Valfre et al., 2008; May, 2009; Dai et al., 2015) and white matter (Szabo et al., 2012; Chong and Schwedt, 2015). However, there have only been a few studies on how the structural parameters of the two sub-groups of migraine differ from each other (Granziera et al., 2013; Rocca et al., 2014). Most studies investigating white matter integrity in migraineurs either found no significant difference between the two groups, used mixed groups, or assessed just one of them (Rocca et al., 2003, 2006; Chong and Schwedt, 2015; Messina et al., 2015). There are, however, several findings that have brought dissimilarities to light, e.g., the diffusion parameters of the visual pathway (Granziera et al., 2006; Rocca et al., 2008) and the thalamocortical tract (DaSilva et al., 2007). These conflicting reports, the incomplete understanding of mechanisms underlying the disorder and the absence of reliable biomarkers urges further research in the area. Previously we showed the alterations of diffusion MRI measured white matter microstructure in a mixed group of migraine patients with and without aura using TBSS (Szabo et al., 2012). In the current investigation, we extended our former results and went on to assess the white matter microstructural alteration in a group of patients with MwA and MwoA. The main goal of this study was to clarify that microstructural changes of the white matter are distinct within subtypes of migraine and to determine whether these alterations are related to clinical parameters.

## Materials and Methods

### Subjects

Eighteen MwA, 25 MwoA were recruited from outpatients of the Headache Outpatient Clinic of the Department of Neurology. The diagnosis was based on the criteria of the International Headache Society (Headache Classification Committee of the International Headache Society, 2013). Hamilton Depression Scale (Hamilton, 1960) was used to screen for depression and patients with more than eight points were excluded from the study. The patients had no other neurological diseases. Twenty-eight controls with no neurological disorders were recruited into the study. The controls were selected to match the age and gender distribution of both patient groups. The Regional Human Biomedical Research Ethics Committee of the University of Szeged approved the study (87/2009). Written informed consent was obtained from all participants.

### MRI Acquisition

All MRI acquisitions were in the interictal period, minimum 1 week after the last headache attack. MRI acquisitions were carried out on a 1.5 T GE Signa Excite HDxt MR Scanner (GE Healthcare, Chalfont St. Giles, United Kingdom). Three dimensional spoiled gradient echo images [FSPGR: echo time (TE): 4.1 ms; repetition time (TR): 10.276 ms; matrix: 256 ^∗^ 256; field of view (FOV): 25 cm ^∗^ 25 cm; flip angel: 15°; in-plane resolution: 1 mm ^∗^ 1 mm; slice thickness: 1 mm] and 60 directions diffusion-weighted images with 6 non-diffusion-weighted reference volume [TE: 93.8 ms; TR: 16.000 ms; matrix: 96 ^∗^ 96; FOV: 23 cm ^∗^ 23 cm; flip angle: 90°; in-plane resolution: 2.4 mm ^∗^ 2.4 mm; slice thickness: 2.4 mm; b: 1000 s/mm^2^; number of excitations (NEX): 2; array spatial sensitivity encoding technique (ASSET) factor = 2] were acquired for all subjects.

### Data Analysis

Image analysis was carried out using tools of FSL (Smith et al., 2004). Diffusion data were corrected for eddy currents and movement artifacts by 12 degrees of freedom affine linear registration to the first non-diffusion-weighted reference image (Jenkinson and Smith, 2001). Diffusion tensors at each voxel were fitted by the algorithm included in the FMRIB’s Diffusion Toolbox of FMRIB’s Software Library [FSL v. 5.0 (Smith et al., 2004)]. Non-brain parts were removed with the brain extraction tool (BET) (Smith, 2002). FA, MD, and AD and RD to the principal diffusion direction were computed for the whole brain.

Tract-based spatial statistics method (Smith et al., 2007) was used to search for the white matter alterations. All subjects’ FA images were aligned into a common space, using the non-linear registration tool, FNIRT, which uses a b-spline representation of the registration warp field. A mean FA image was created and the threshold set at FA = 0.2, deriving a mean FA skeleton that represented the centers of all tracts common to the group. Each subjects’ aligned FA data were then projected onto this skeleton and the resulting data fed into voxel-wise cross-subject statistics. Modeling and inference using standard GLM design set-up was accomplished using permutation-based cluster analysis (*n* = 5000) as implemented in the FSL software package (Nichols and Holmes, 2002). The regressors of the GLM analysis coded for group membership or clinical variables. Three consecutive analyses were conducted for group comparisons: MwA vs. controls, MwoA vs. controls and MwA vs. MwoA. Correlation analysis was conducted between diffusivity parameters and disease duration and attack frequency. The regressors (disease duration and attack frequency) were demeaned. With the GLM design negative and positive correlation were calculated. Statistical thresholding was carried out with Threshold Free Cluster Enhancing (Smith and Nichols, 2009).

*p*-Values less than 0.05 corrected was accepted as significant result. Statistical maps were thresholded for 0.05 and the Johns Hopkins University white-matter atlas was used to identify the anatomical locations of altered regions.

## Results

### Clinical Variables

The clinical and demographic variables of the patients and the control group are summarized in **Table 1**. No significant differences were found between the age or gender distribution of the groups (*p* > 0.05). The patients’ groups didn’t differ in disease duration (*p* > 0.05) or attack frequency (*p* > 0.05).

**Table 1:** Demographic and clinical data.

	MwA (*n* = 18)	MwoA (*n* = 25)	Controls (*n* = 28)
Age, years ± (*SD*)	32.11 ± 8.01	35.69 ± 8.61	31.74 ± 9.58
Male (*n* =)	3	3	3
Disease duration, years ± (*SD*)	14.89 ± 8.45	12.76 ± 9.97	N.A.
Headache frequency, attack/year ± (*SD*)	29.03 ± 25.31	46.22 ± 33.48	N.A.
Visual aura	17	N.A.	N.A.

### Group Differences in White Matter Microstructure

#### MwA vs. Controls

**FIGURE 1:**
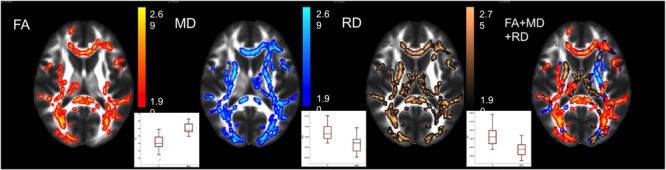
White matter abnormalities in MwA compared to controls. Axial slices show the diffusivity parameter changes from TBSS. The FA increased, mean and radial diffusivity decreased in migraine aura compared to controls. Axial diffusivity showed no alteration. The affected areas are mainly overlapping (see in 4th brain). The color bar shows the z-scores of the corrected *p*-values. The boxplot shows the MD parameters depicted from the affected areas, the central mark is the median and the boxes represent the 25 and 75% percentiles.

Decreased RD (*p* < 0.036; peak Z score = 4.545) was found in MwA compared to controls in the corpus callosum, bilaterally in the parieto-occipital and in the cingular white matter. There was a trend showing decreased MD (*p* < 0.068; peak Z score = 3.541) in MwA in overlapping areas and increased FA (*p* < 0.061) in the corpus callosum (**Figure 1**).

#### MwoA vs. Controls

No significant alterations were found between MwoA and the control group.

#### MwA vs. MwoA

**FIGURE 2:**
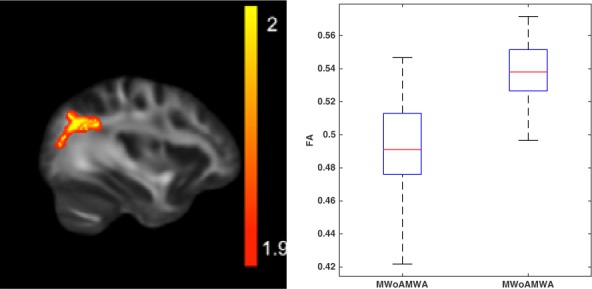
White matter alterations in MwA compared to MwoA. Tract-TBSS indicate increased FA in MwA compared to migraine without. The color bar shows the z-scores of the corrected *p*-values. The boxplot shows the MD parameters depicted from the affected areas, the central mark is the median and the boxes represent the 25 and 75% percentiles.

Higher FA (*p* < 0.048; peak Z score = 3.974) was found in MwA in the left parieto-occipital white matter (**Figure 2**).

### Correlation of Clinical Variables with Diffusion Parameters

**FIGURE 3:**
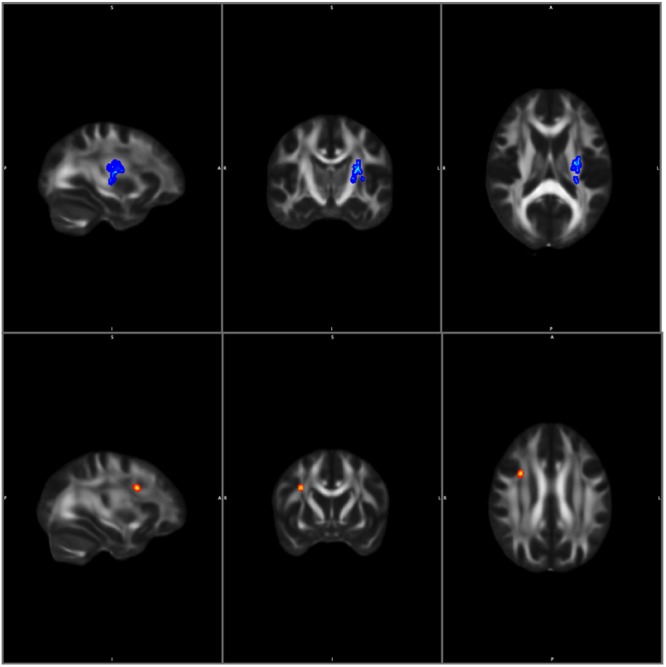
Correlation with clinical parameters in MwA. Blue color shows negative correlation with disease duration and red color shows negative correlation with attack number to axial diffusivity. Statistical images are thresholded at *p* < 0.05 corrected for multiple comparisons.

In MwA, the AD negatively correlated with disease duration in the left superior longitudinal fascicle (left parieto-occipital region) and in the left corticospinal tract (*p* < 0.036; peak Z score = 5.765). The estimated lifetime attack number showed a negative correlation to the AD in the right superior longitudinal fascicle (*p* < 0.048; peak Z score = 5.621) in the MwA group (**Figure 3**). There was no significant correlation of the clinical variables in the MwoA group.

## Discussion

In this study we provided evidence for interictal white matter microstructural alterations in MwA, a difference not appearing in MwoA patients.

In our very recent study, the amplitude of the resting state functional MRI activity fluctuation was higher in MwA compared to MwoA (Farago et al., 2017). One possible source of the difference between the two subgroups of migraine patients could be the presence of CSD in MwA. Functional imaging studies showed the signature of the slow depolarization wave spreading over the cortex during aura phase (Hadjikhani et al., 2001). No similar sign of slow depolarization was detected in MwoA patients. CSD induces the release of neurotransmitters that leads to neuroinflammation, glial cell activation (Charles and Brennan, 2009) and oxidative stress (Shatillo et al., 2013). Moreover, there is evidence that CSD modifies circulation in the brain, altering its susceptibility to ischaemia (Ayata and Lauritzen, 2015). All these processes might result in white matter abnormalities. However, while molecular markers of cell damage such as S100B and neuron-specific enolase can be detected in the serum of migraine patients (Teepker et al., 2009; Yilmaz et al., 2011), no difference was found between MwA and MwoA patients that could reflect the structural damage that we have found.

Based on the typical clinical course of the symptoms during the aura, it is thought that the CSD originates from the occipital cortex and spreads over the most of the post-central brain (Petrusic and Zidverc-Trajkovic, 2014). In our study, the signature of altered microstructure was found in the inferior fronto-occipital fasciculus, thalamo-cortical tracts and the corpus callosum of MwA as compared to controls. Differences between MwA and MwoA patients were detected in the occipito-parietal region that might be coherent with the proposed spread of CSD during the aura phase. Although, it cannot fully explain the topical nature of the differences seen between MwA and MwoA. CSD in MwoA may affect silent areas in the brain and therefore no aura symptoms develop (Ayata, 2010; Cosentino et al., 2014). Furthermore, we cannot exclude that the detected structural alterations are related to the fact that CSD is causing robust changes to brain circulation (Lauritzen, 1987).

Another possible option behind the microstructural alterations might be the cortical hyperexcitability in migraine (Gawel et al., 1983; Aurora et al., 1998; Afra et al., 2000; Antal et al., 2005; Chadaide et al., 2007; Pierelli et al., 2013). As was shown in a recent meta-analysis, the TMS-evoked phosphene threshold is lower in MwA and the prevalence of phosphenes was also higher in MwA (Brigo et al., 2012). Cross-modal perception in MwoA and MwA suggests abnormal cortical excitability, which is more affected in MwA patients (Brighina et al., 2015). Recent reports also pointed out that the visual evoked potential measured hyperexcitability predominantly true for MwA (Sand et al., 2008; Coppola et al., 2015).

It is disputed whether there is an alteration in white matter microstructure in MwA and MwoA. In a recent study, Tedeschi et al. (2016) detected no difference in diffusivity parameters in MwA and MwoA. An earlier study by Granziera et al. (2006) found no difference between MwA and MwoA groups using voxel-based morphometry-style analysis of diffusion parameters. In our previous study in a mixed group of episodic MwA and MwoA, we presented a relatively smaller pre-frontal white matter alteration (Szabo et al., 2012). Similarly to our study, using TBSS approach, diffusivity parameter changes were published in MwoA (Yuan et al., 2012; Yu et al., 2013). Neeb et al. (2015) found no DTI changes in chronic and episodic MwoA. These studies are consistent in finding reduced FA in the white matter of patients with MwoA. In our cohort, there was no significant difference in the white matter diffusion parameters of MwoA and controls, like in Tedeschi’s paper (Tedeschi et al., 2016). However, the MwA patients showed a significantly higher FA in extensive white matter regions, which differs from that study. The importance of the number of the diffusivity directions and how the tensors are calculated is underlined by the literature (Barrio-Arranz et al., 2015). We used the same approach to analyze the DTI data, but Tedeschi’s group measured DTI with 32-diffusivity direction once and in our study, we used 60 directions DTI measured twice which might account for the different results. Additionally, the variant MRI parameters (Landman et al., 2007) used in clinical studies and the number of gradient directions have measurable effects on estimated parameters (Barrio-Arranz et al., 2015).

In our previous paper we proposed two alternative hypotheses to explain the white matter microstructural changes: (i) repeated painful conditions or increased cortical excitability might cause maladaptive plastic changes or alternatively (ii) cortical hyperexcitability and CSD might cause degenerative changes through glutamatergic excitotoxicity and mitochondrial dysfunction (Longoni and Ferrarese, 2006; Moskowitz, 2007; Sas et al., 2010). It seems that in MwA patients the first alternative is dominant, represented by increased FA. Use-dependent plasticity-related gray matter morphological alterations, together with the white matter microstructural changes, were reported earlier (Draganski et al., 2004; Boyke et al., 2008; Teutsch et al., 2008; Scholz et al., 2009; Lerch et al., 2011). The underlying mechanism is still disputed. In learning, where repeated stimuli as “mind training” occur, it is thought to be related to the locally enhanced myelination that is represented by an increase in FA (Wedeen et al., 2005; Blumenfeld-Katzir et al., 2011; Lerch et al., 2011; Sampaio-Baptista et al., 2013). Recently, it was suggested that a correlation exists between nerve conduction velocity and locally measured FA (Akhtari et al., 2006; Brienza et al., 2014; Heckel et al., 2015; Wang et al., 2015). We hypothesize that repeated painful attacks or cortical hyperexcitability induce used-dependent plasticity in MwA patients, which in the white matter is represented by increased FA, a signature of a more compact structure.

The longer the history of migraine, the lower the white matter AD. This finding is similar to that described by Yu et al. (2013). Altered AD was reported in axonal loss (Pierpaoli et al., 2001; Kim et al., 2006; Sun et al., 2006) and molecular markers also point to neuronal and glial damage in migraine (Yilmaz et al., 2011). A negative correlation between disease duration and AD might be the sign of chronic myelin and axonal damage (Kincses et al., 2013; Benitez et al., 2014; Gregory et al., 2015), such as in neurodegenerative disorders. Another explanation might be that migraine pathology doesn’t affect the brain areas equally and some pathways become more robust due to plasticity.

## Conclusion

Migraine is a heterogeneous disease and our results indicate that degenerative and maladaptive plasticity coexist in the disease. The variable diffusion profiles described in the current study and earlier investigations are a consequence of these processes. Our results also point to a possible difference in the pathomechanism of MwA and MwoA, suggesting a separate investigation of these two forms.

## Author Contributions

LV, ZTK, and NS planned the project and formulated the study hypothesis. ÁP, JT, and DS recruited the patients. BK, GC, ET, and KK organized and carried out the MRI measurements. BT, DV, and NS collected the clinical data. AK, PF, and NS analyzed the MRI data and carried out the statistical analysis. NS,DV, ZTK, ÁP, DS, and JT formalized the discussion of the results. NS, ZTK, DV, LV, and ÁP wrote the manuscript.

## Conflict of Interest Statement

The authors declare that the research was conducted in the absence of any commercial or financial relationships that could be construed as a potential conflict of interest.

## Abbreviations

AD: diffusivity parallel
CSD: cortical spreading depression
DTI: diffusion tensor imaging
FA: fractional anisotropy
FSL: FMRIB Software Library
GLM: general linear model
MD: mean diffusivity
MwA: migraine with aura
MwoA: migraine without aura
RD: perpendicular
TBSS: tract-based spatial statistics
